# Comparative profiling of white matter development in the human and mouse brain reveals volumetric deficits and delayed myelination in Angelman syndrome

**DOI:** 10.21203/rs.3.rs-4681861/v1

**Published:** 2024-08-09

**Authors:** Siddhi S. Ozarkar, Ridthi K.-R. Patel, Tasmai Vulli, Audrey L. Smith, Mark D. Shen, Alain C. Burette, Benjamin D. Philpot, Martin A. Styner, Heather C. Hazlett

**Affiliations:** Neuroscience Center, University of North Carolina at Chapel Hill, Chapel Hill, NC; Department of Cell Biology and Physiology, University of North Carolina at Chapel Hill, Chapel Hill, NC; Neuroscience Center, University of North Carolina at Chapel Hill, Chapel Hill, NC; Department of Cell Biology and Physiology, University of North Carolina at Chapel Hill, Chapel Hill, NC; Neuroscience Center, University of North Carolina at Chapel Hill, Chapel Hill, NC; Department of Cell Biology and Physiology, University of North Carolina at Chapel Hill, Chapel Hill, NC; Neuroscience Center, University of North Carolina at Chapel Hill, Chapel Hill, NC; Department of Cell Biology and Physiology, University of North Carolina at Chapel Hill, Chapel Hill, NC; Neuroscience Center, University of North Carolina at Chapel Hill, Chapel Hill, NC; Carolina Institute for Developmental Disabilities, University of North Carolina at Chapel Hill, Chapel Hill, NC; Department of Psychiatry, University of North Carolina at Chapel Hill, Chapel Hill, NC; Neuroscience Center, University of North Carolina at Chapel Hill, Chapel Hill, NC; Department of Cell Biology and Physiology, University of North Carolina at Chapel Hill, Chapel Hill, NC; Neuroscience Center, University of North Carolina at Chapel Hill, Chapel Hill, NC; Department of Cell Biology and Physiology, University of North Carolina at Chapel Hill, Chapel Hill, NC; Carolina Institute for Developmental Disabilities, University of North Carolina at Chapel Hill, Chapel Hill, NC; Carolina Institute for Developmental Disabilities, University of North Carolina at Chapel Hill, Chapel Hill, NC; Department of Psychiatry, University of North Carolina at Chapel Hill, Chapel Hill, NC; Department of Computer Science, University of North Carolina at Chapel Hill, Chapel; Carolina Institute for Developmental Disabilities, University of North Carolina at Chapel Hill, Chapel Hill, NC; Department of Psychiatry, University of North Carolina at Chapel Hill, Chapel Hill, NC

## Abstract

**Background:**

Angelman syndrome (AS), a severe neurodevelopmental disorder resulting from the loss of the maternal *UBE3A* gene, is marked by changes in the brain’s white matter (WM). The extent of WM abnormalities seems to correlate with the severity of clinical symptoms, but these deficits are still not well characterized or understood. This study provides the first large-scale measurement of WM volume reduction in children with AS. Furthermore, we probed the underlying neuropathology by examining the progression of myelination in an AS mouse model.

**Methods:**

We conducted magnetic resonance imaging (MRI) on children with AS (n=32) and neurotypical controls (n=99) aged 0.5–12 years. In parallel, we examined myelination in postnatal *Ube3a* maternal-null mice (*Ube3a*^m−/p+^; AS model), *Ube3a* paternal-null mice (*Ube3a*^m+/p−^), and wildtype controls (*Ube3a*^m+/p+^) using immunohistochemistry, Western blotting, and electron microscopy.

**Results:**

Our data revealed that AS individuals exhibit significant reductions in brain volume by ~1 year of age, with WM reduced by 26% and gray matter by 21% by 6–12 years of age—approximately twice the reductions observed in the adult AS mouse model. In our AS mouse model, we saw a global delay in the onset of myelination, which normalized within days (likely corresponding to months or years in human development). This myelination delay is caused by the loss of UBE3A in neurons rather than UBE3A haploinsufficiency in oligodendrocytes. Interestingly, ultrastructural analyses did not reveal any abnormalities in myelinated or unmyelinated axons.

**Limitations:**

It is difficult to extrapolate the timing and duration of the myelination delay observed in AS model mice to individuals with AS.

**Conclusions:**

This study reveals WM deficits as a hallmark in children with AS, demonstrating for the first time that these deficits are already apparent at 1 year of age. Parallel studies in a mouse model of AS show that these deficits may be associated with delayed onset of myelination due to the loss of neuronal (but not glial) UBE3A. These findings emphasize the potential of WM as both a therapeutic target for interventions and a valuable biomarker for tracking the progression of AS and the effectiveness of potential treatments.

## Background

Angelman syndrome (AS, OMIM #105830) is a severe neurodevelopmental disorder that manifests within the first year of life. Individuals with AS exhibit severe intellectual disability, developmental delay, speech impairment, motor dysfunction, behavioral abnormalities, microcephaly, sleep disturbances, seizures, and abnormal EEG patterns. [1, 2, 3, 4, 5, 6]. The first signs of developmental delay typically appear between 6 and 12 months of age, with additional symptoms gradually emerging throughout early childhood.

AS arises from loss-of-function mutations or deletions in the maternal *UBE3A* gene encoding a HECT domain E3 ubiquitin ligase (UBE3A, OMIM# 601623). Although UBE3A is expressed in all tissues, its expression is regulated by genomic imprinting. In most cells, both the maternal and paternal copies of the *UBE3A* gene are active. However, in neurons, the paternal *UBE3A* allele is transcriptionally silenced by an antisense transcript [7, 8, 9, 10]. This monoallelic expression makes neurons uniquely vulnerable to maternal *UBE3A* loss-of-function mutations or deletions. Conversely, in non-neuronal cells such as astrocytes and oligodendrocytes, both parental *UBE3A* alleles are transcribed. This dual expression potentially mitigates the impact of maternal *UBE3A* deficiencies. Despite the identification of numerous potential UBE3A protein substrates and interacting partners, and the established role of UBE3A in synaptic plasticity [11], the precise mechanisms through which UBE3A loss in neurons cause the pervasive clinical manifestations of AS remain poorly understood.

Neuroimaging studies in children with AS reveal significant reductions in brain volume, widespread alterations in white matter (WM), and global decreases in both functional and structural connectivity [12, 13, 14, 15, 16]. Importantly, greater WM deficits correlate with more severe phenotypic expression in AS [15, 16, 17]. Similarly, we found a disproportionate reduction in WM compared to gray matter (GM) loss in our AS model mice [18]. Taken together, these observations underscore the need to better characterize aberrant WM development in AS, especially in the postnatal brain, when WM development is most dynamic [19] and AS symptoms start to appear. A better understanding of WM development in AS will enhance our knowledge of AS pathology and aid in developing therapies to mitigate WM deficits.

To investigate the consequences of maternal *UBE3A* loss on WM development after birth, we (1) characterize the postnatal trajectory of WM volume in children with AS (aged 1–12 years) and (2) examine differences in myelination patterns in postnatal AS model mice compared to wild-type (WT) controls.

## Methods

### Human MRI

We conducted MRIs on N = 131 children between 0.5–12 years of age, n = 32 children with AS and n = 99 neurotypical (NT) control participants. Total brain volumes (TBV) were generated in all scans acquired between 0.5–12 years. White matter (WM) and gray matter (GM) volumes were generated in the subset of scans acquired between 1–12 years, owing to the ability to automatically segment between WM/GM tissues in MRI scans acquired over age 1 year. The final sample in the WM and GM analyses included N = 94 children: n = 28 AS (19M, 9F) and n = 66 age- and sex-matched NT controls (54M, 12F). Of these, 25 participants (13 AS; 12 NT) had a second longitudinal MRI scan, yielding a total of 119 scans in the WM and GM analyses (41 AS scans; 78 NT scans). T1- and T2-weighted structural MRI scans (1mm3 voxels) were acquired on 3T Siemens TIM Trio scanners with a 12-channel head coil. T1 and T2 images underwent registration, transformation to stereotactic space, and segmentation of total brain, WM, and GM volumes [20, 21, 22].

AS participants were confirmed with a chromosomal microarray showing a chromosomal deletion of the 15q11.2–13 region on the maternal allele. NT participants were enrolled and had no first-degree relatives with a psychiatric diagnosis [23]. All subjects were excluded for the presence of: (a) diagnosis or physical signs of known genetic conditions or syndromes other than AS (e.g., significant dysmorphology, asymmetry on physical exam), significant medical or neurological conditions affecting growth, development or cognition (e.g., CNS infection, diabetes, tuberous sclerosis, congenital heart disease), or sensory impairments such as significant vision or hearing loss (or evidence of during the course of the study); (b) a history of significant perinatal adversity, exposure to in-utero neurotoxins (including alcohol, illicit drugs, selected prescription medications), or a history of maternal gestational diabetes; (c) contraindication for MRI (pacemaker, vascular stents, metallic ear tubes, other metal implants or braces); (d) families whose predominant home language is not English; and (e) children who were adopted. Parents of AS and NT individuals provided informed consent, and the institutional review board approved the research protocol.

### Animals

AS model mice (*Ube3a*^m–/p+^), which maternally inherit the *Ube3a* knock-out allele, were generated by mating wild-type (*Ube3a*^m+/p+^) male mice to female mice with paternal inheritance of the *Ube3a* knock-out allele (*Ube3a*^m+/p−^, paternal null model), which themselves are phenotypically normal.

### Antibodies

To identify myelin basic protein (MBP), we used a rat monoclonal antibody (Abcam Cat#ab7349, RRID: AB_305869) raised against the full-length protein corresponding to cow MBP. This antibody binds to a region defined by amino acids 82–87 (DENPVV).

### Western Blotting

Animals were anesthetized with isoflurane, followed by decapitation and extraction of forebrain tissue, excluding the olfactory bulb, cerebellum, and brainstem. The tissue was homogenized in a lysis buffer containing Tris-HCl (25mM, pH 7.4), 1% SDS, and 1mM EDTA with protease inhibitor. Protein concentrations were determined with the BCA assay, and protein (30 μg for P14 and 20 μg for P45) was loaded onto a 20% polyacrylamide-SDS gel. Proteins were then transferred onto a 0.2 μm nitrocellulose membrane. After blocking with Intercept Blocking Buffer (LiCor) for 1 hr, the membranes were incubated overnight at 4°C with primary antibodies: rat anti-MBP (1:1,000) and mouse anti-GAPDH (1:5,000; Sigma Cat#MAB374; RRID:AB_2107445). Following washing with PBS-Tween (PBS with 0.1% Tween^™^ 20), membranes were incubated in HRP-conjugated secondary antibodies, washed in PBS-Tween, and chemiluminescence imaging was performed. MBP protein band intensity was normalized to GAPDH band intensity. Protein expression in WT and *Ube3a* mutants was represented as a fraction of protein level in WT mice.

### Light microscopy

Mice were anesthetized with sodium pentobarbital (60 mg/kg i.p.) and subsequently transcardially perfused, starting with a rapid flush with PBS (0.1 M, pH 7.3), followed by 10 mins of 4% freshly depolymerized paraformaldehyde in phosphate buffer (pH 7.3). Brains were then postfixed overnight at 4°C in the same fixative solution, cryoprotected in 30% sucrose in PBS, and sectioned at 50 μm using a sliding microtome. Free-floating sections underwent an initial methanol permeabilization step (2 × 15 mins in 50% methanol in PBS) followed by a second permeabilization step: 1 hour at 37°C in a solution comprising 2.3% Glycine, 20% DMSO, and 0.2% Triton X-100 in PBS. Subsequently, sections were preincubated for 30 minutes in 5% DMSO/0.1% Triton X-100/1% BSA in PBS and incubated overnight at 37°C with primary antibody (MBP, 1:2,000 in PBS with 5% DMSO, 0.1% Triton X-100, 1% BSA, 0.2% Tween-20, and 1% heparin). The primary antibody was visualized using a secondary antibody conjugated with Alexa Fluor dye. Sections were counterstained with DAPI to reveal nuclei, and scanning was performed using a Slideview VS200 slide scanner (Olympus, Hamburg, Germany). Analysis was conducted using the QuPath software package (Bankhead et al., 2017).

### Electron microscopy

Mice were anesthetized with sodium pentobarbital (60 mg/kg i.p.) and perfused with a solution containing 2% glutaraldehyde, 2% paraformaldehyde, and 0.2% picric acid in 0.1 M phosphate buffer (pH 6.8). Following perfusion, brains were promptly removed and postfixed overnight at 4°C in the same fixative. Subsequently, the brains were sectioned to a thickness of 50 μm using a vibratome. The sections were processed for reduced osmium following the Knott protocol [24, 25, 26]. In brief, the sections were washed in cacodylate buffer (0.1 M, pH 7.4) and post-fixed for 40 minutes in a solution of 1.5% potassium ferrocyanide and 1% osmium tetroxide, followed by an hour in 1% osmium tetroxide alone. After rinsing in water, the sections were incubated for 40 minutes in 1% uranyl acetate in water, rinsed again in water, dehydrated in an ethanol series, and finally infiltrated and embedded in resin (Spurr’s low viscosity epoxy with ERL-4221, Electron Microscopy Sciences, Hatfield, PA; cat. No. 14300). The embedded sections were flat mounted between sheets of ACLAR^®^ fluoropolymer (Electron Microscopy Sciences, Hatfield, PA; cat. No. 50425) within glass slides. Small chips of the corpus callosum (body region) were affixed to plastic blocks, sectioned *en face* at ~ 60 nm, collected on 300 mesh nickel grids, and coated and contrasted with uranyl acetate and Sato’s lead. Grids were imaged at a voltage of 120 kV using a Technai 12 D230 transmission electron microscope running SerialEM [27, 28].

We acquired large 50–60 μm by 50–60 μm montages at 6500x (close to 1nm per pixel) for quantification. Axons and myelin were manually traced using FIJI [29, 30]. The relative myelin thickness around an axon (*g*-ratio) was calculated as the √(AxonArea/MyelinArea).

### Experimental Design and Statistical Analysis

Differences in human MRI brain volumes (TBV, WM, GM) between the AS and NT groups were tested using a mixed effects model for repeated measures while covarying for the fixed effects of age, sex, scanner, and group x age interaction. Random effects included the individual subjects’ age at scan. All statistical analyses of MRI data were performed using SAS JMP software.

Early nutrition and maternal care are known to influence brain development, specifically growth and myelination. To account for these factors, we sampled AS mice and their WT littermates at various ages, ensuring litters were culled to a size of 5–7 mice. Sample sizes varied by experiment: for light microscopy, a minimum of 3 pairs of AS and WT littermates were examined across P2 through P60; transmission electron microscopy used 4 mice of each genotype at P16 and P30; Western blotting analyzed 10 pairs of WT and AS mice at P14, 7 pairs of WT and paternal *Ube3a*-null model (*Ube3a*^m+/p−^, PNL) mice at P14, and 7 pairs of WT and AS mice at P45. Statistical analyses were performed using GraphPad Prism 9 (RRID:SCR_002798). These included unpaired two-tailed t-tests for MBP protein levels and percentage of myelinated axons.

## Results

Our previous MRI studies showed decreased white matter (WM) volume in adult AS model mice. To investigate whether this finding extends to children with AS and to explore its developmental trajectory, we conducted MRIs on children with AS compared to NT controls (Fig. 1). Representative MRIs from an AS and NT individual are depicted in Fig. 1A–B, with total brain volume (TBV) segmented into WM and GM volumes. As hypothesized, AS children had significantly smaller TBV from 0.5–12 years compared to NT children (F1,234 = 28.82, p < .0001, covarying for age, sex, scanner, group x age; Fig. 1C). This smaller TBV in AS was comprised of significantly smaller WM volumes from 1–12 years compared to NT controls (F1,103 = 24.46, p < .0001, covarying for age, sex, scanner, group x age; Fig. 1D) and GM volumes (F1,99 = 34.98, p < .0001; Fig. 1E). The lack of group x age interaction in WM trajectories (p = 0.42) indicated that the AS group had significantly smaller WM volumes at all ages from 1 to 12 years (Fig. 1D).

The neurotypical trajectory of TBV shows rapid growth from 6 months to around 6 years of age, at a rate of more than 10% per year (Fig. 1C). After 6 years of age, TBV growth continues but at a slower rate of ~ 4% per year. During this period between 6–12 years of age, when the rate of brain growth has slowed and is more stable (i.e., the dotted rectangles in Fig. 1D–E), we calculated the magnitude of difference between the AS and NT groups in WM and GM volumes (controlling for covariates). The decreased TBV in AS was driven by a 26.5% decrease in WM volume (Fig. 1F) and a 21.6% decrease in GM volume (Fig. 1G) compared to NT controls.

Given the correlation between WM deficits and the severity of behavioral issues [15, 16, 17], we focused on understanding WM reductions using our AS mouse model. Since myelination plays a critical role in the increase of WM volume during postnatal development, we compared the degree of myelination in WT and AS mouse brains at two crucial developmental stages: P14, which is a peak period of active myelination, and P45, by which time myelination is mainly complete. We used MBP as a marker to gauge myelination levels. Western blot analysis at P14 revealed approximately 20% less MBP in the forebrains of AS mice than in WT controls (Fig. 2A, B). This reduction in overall MBP was primarily due to the decreased levels of the two exon-II-containing isoforms, 21.5 kDa and 17.22 kDa, which are known to play a role in early, active myelination [31, 32, 33, 34].

Interestingly, this lower level of MBP was not seen in mice that lacked the paternal *Ube3a* allele (Fig. 2C, D). Paternal *Ube3a*-null mice (*Ube3a*^m+/p−^), in contrast to AS model mice (*Ube3a*^m−/p+^), retain UBE3A protein expression in neurons, while oligodendrocytes in both the maternal- and paternal-null models express only one functional *Ube3a* copy. This finding suggests that the MBP expression deficit seen in AS arises from the loss of UBE3A in neurons rather than haploinsufficiency of *Ube3a* within oligodendrocytes. Surprisingly, the MBP differences between groups at P45 did not reach statistical significance (p = 0.41, Fig. 2E, F), suggesting that AS mice experience a delay in myelination rather than a permanent deficit.

To better understand myelination patterns in AS model mice, we used immunohistochemistry to compare regional MBP distribution in WT and AS postnatal brains (Fig. 3). In both WT and AS brains, the developmental distribution of MBP, indicative of myelination, follows the well-established caudo-rostral myelination pattern. MBP staining begins in the spinal cord and progresses systematically through the brainstem regions (medulla, pons, mesencephalon) and finally into the telencephalon. However, AS mice show a consistent delay of several days in the appearance and intensity of MBP staining compared to their age-matched WT littermates. This delay is especially clear during the initial stages of myelination, with the timing varying across different brain regions.

For example, in the superior olivary complex at P8, AS mice displayed a reduced density of MBP-positive fibers compared to WT mice (Fig. 4). However, by P10, this difference in staining density was no longer noticeable. In the WT cerebellum at P8, the granule cell layer displayed abundant MBP-positive premyelinating oligodendrocytes, characterized by many delicate, radiating processes resembling a spider’s web. A few MBP-positive fibers were also visible in the lower part of the granule cell layer. In sharp contrast, the AS cerebellar cortex at this stage completely lacked MBP staining (Fig. 5). By P12, the granule cell layer in the WT brain showed a progression in myelination, with increased MBP-positive fibers and longer processes reaching toward the Purkinje cell layer, but this progression was less pronounced in the cerebellar cortex of AS mice, which exhibited fewer fibers and shorter processes. This delay in myelination persisted at P28 but was unnoticeable by P45.

The late onset of myelination in AS mice was clearest in the motor-related part of the superior colliculus at P8 (Fig. 6). In WT mice, MBP staining at P8 indicated the onset of myelination (Fig. 6 inset in P8 WT micrograph). In contrast, in AS mice at the same age, MBP staining was localized to cells displaying morphological features of premyelinating oligodendrocytes (Fig. 6 inset in P8 AS micrograph). MBP staining in the colliculi of the AS brains continued to exhibit reduced density at both P16 and P28, although this difference was normalized by P60.

Myelination in the hippocampal area also experienced delays; while in the dentate gyrus the delay normalized by P60, in the CA1 region, AS littermates still showed reduced MBP signal, albeit modestly (Fig. 7, 8). WM pathways in AS mice also showed myelination delays, including fibers penetrating the globus pallidus (Fig. 9) and the corpus callosum (Fig. 10). At P5, MBP staining in the globus pallidus of WT mice showed early-stage myelination, while, in AS mice, it was predominantly localized to premyelinating oligodendrocytes (Fig. 9). This delay persisted until P10 but was resolved by P12. In the body and genu of the corpus callosum, AS mice showed a reduced density of MBP-positive fibers compared to WT mice at P16 (Fig. 10). While the MBP signal in the body region normalized by P30, the genu did not normalize until P60.

Immunohistological data confirmed the Western blot findings, revealing a delay in the onset of myelination across all brain regions examined, but that was ultimately normalized. This raises the question of whether this delay is associated with ultrastructural anomalies. To investigate this, we employed transmission electron microscopy, focusing on the body of the corpus callosum (Figs. 11, 12). We examined two-time points: P16, when immunohistochemistry in the body of the corpus callosum revealed a clear myelin deficit, and P30, when the deficit appeared normalized. Ultrastructural analysis showed no evidence of axonopathy in either genotype at any age (Fig. 11). At P16, both genotypes exhibited a typical range of myelination stages: initial myelin extension, ongoing wrapping and compaction, and axons with fully compacted myelin. By P30, while axons still presented early and intermediate stages of myelination, there was a notable shift towards more mature myelin. Quantitative analysis revealed a significant delay in myelination in AS model mice at P16, with about 35% fewer myelinated axons than their WT littermates (Fig. 12). However, by P30, this difference was no longer significant. The average diameter of both unmyelinated and myelinated axons in WT and AS mice did not differ significantly at either age. Additionally, we did not see differences in the relative thickness of the myelin sheath (*g*-ratio) between WT and AS groups at any age.

Taken together, our data support a model of delayed myelination onset in AS mice rather than a fundamental defect in the myelination process itself.

## Discussion

Postnatal microcephaly is a common clinical feature of children with AS and is typically noticed by the age of two. However, it may be present earlier but go unnoticed due to the limitations of imaging technology. Like AS individuals, AS mouse models also show microcephaly [18]. Our previous MRI studies on these mouse models indicate that a reduction in WM volume is an important contributor to microcephaly in AS [18]. This follow-up study shows that a significant WM deficits are also present in children with AS. Our MRI data show a ~ 26% reduction in WM volume in AS children aged 6–12 years. This confirms that altered WM throughout the brain is a key feature of AS, expanding on earlier findings [12, 13, 14, 15, 16]. Interestingly, we find a greater reduction in AS individuals compared to the AS mouse model. Specifically, AS individuals showed a WM decrease of around ~ 26%, whereas the mice only showed an 11–13% reduction [18]. Similarly, GM volume was reduced by ~ 21% in AS individuals compared to 7–8% in the mouse model [18]. Whether these differences stem from species-specific differences in UBE3A biology or idiosyncratic anatomical variations between rodents and primates is still an open question.

WM is fundamental to infant behavior, with healthy development linked to cognitive function, visual attention, working memory, and language skills [35, 36, 37, 38, 39]. Conversely, atypical WM development is a hallmark of several neurodevelopmental diseases [40, 41]. Therefore, the significant reduction in WM volume seen in this study is likely an important contributor to AS pathology. However, the remarkable plasticity of WM during development also presents a promising avenue for AS treatment. Recent research highlights how the frequency of parent-infant conversations can directly affect the myelination of key brain pathways [42]. This plasticity suggests that targeted therapies focused on enhancing WM health could lead to significant improvements for individuals with AS.

Several promising treatments aimed at restoring UBE3A function are currently undergoing clinical trials for AS [43]. Considering the large WM deficit seen in this study, its link to behavioral traits in AS [15, 16, 17], and the plasticity of WM, measures of WM integrity hold promise as therapeutic biomarkers. Current imaging methods struggle to accurately distinguish between WM and GM in infants under one year old because of lower levels of myelination, which hampers the early detection of WM deficits. Nevertheless, rapid advancements in computer vision technology may soon overcome this limitation, thereby increasing the effectiveness of WM as a therapeutic biomarker.

The microanatomical basis of the reduction in WM volume detected by MRI is still unclear. Several factors may contribute, including myelination deficits, reduced axonal density or diameter, or abnormal organization of axons. Our data from the AS mouse model showed a temporary delay in the onset of myelination, which quickly resolves, resulting in a normalized percentage of myelinated axons by P30. Myelinated axons in the AS brain displayed a normal *g*-ratio, indicating a typical axon diameter to myelin thickness ratio, and were indistinguishable from their counterparts in the WT brain. Our earlier studies suggested a decrease in axon size [18]. However, in this study, we did not see any significant reduction in axon diameter, either in myelinated or unmyelinated axons. This discrepancy might be attributed to multiple factors: the inherent spatial heterogeneity within the corpus callosum, the possibility that UBE3A loss has region-specific effects, and age differences. Finally, we saw no overt defects in the myelination process or any ultrastructural abnormalities within the WM, leaving the WM volume deficit unexplained. However, the timing of myelination is critical as myelin stabilizes axon structure and wiring patterns in neural networks, suggesting that even transient delays could lead to enduring changes in brain connectivity [44]. Indeed, AS individuals have significantly reduced overall connectivity [16, 45]. It is also worth noting that while we saw a brief delay in myelination in mice (days), this likely translates to a more extended period in humans (potentially months or even years). An extended delay in humans could have a significantly greater impact on AS brain development. It might explain the greater WM deficit we observed in AS individuals compared to mouse models. On the positive side, this also implies extended windows for therapeutic intervention.

The exact mechanism behind the observed delay in myelination remains unclear, although our data offer some insights. UBE3A is expressed in oligodendrocytes [10] and likely in oligodendrocyte precursor cells. Theoretically, the maternal loss of UBE3A in oligodendrocytes, which express *Ube3a* biallelically, could impair their ability to myelinate, leading to a delay in myelination. It has yet to be definitively established whether *Ube3a* haploinsufficiency in oligodendrocytes leads to a 50% reduction in UBE3A protein levels. However, our results from AS model mice show that *Ube3a* haploinsufficiency in oligodendrocytes does not lead to the observed myelination deficits, as this delay is not present in our paternal *Ube3a*-null model mice. Thus, our data suggest that the delayed myelination is associated with the absence of UBE3A in neurons. Neurons can influence myelination through various mechanisms [46, 47], and the loss of UBE3A could disrupt one or more of these processes, resulting in a delay in myelination. Several secreted molecules from active neurons have been reported to increase oligodendrocyte precursor cell proliferation and/or differentiation, including PDGF [48], brain-derived neurotrophic factor [49], adenosine 5′-triphosphate [50], and glutamate [51, 52].

Oligodendrocyte precursor cells also express neurotransmitter receptors and can be directly regulated by synaptic transmission [53]. Therefore, neuronal activity can regulate the early steps of myelin formation, including oligodendrocyte precursor cell proliferation and differentiation, to affect overall myelination. Consequently, a delay in neuronal maturation could lead to a delay in myelination. This highlights the importance of further investigating neuronal development in AS, especially given our findings that GM volume is also reduced in children with AS aged 1–12, albeit to a lesser degree than WM volume.

## Limitations

Assessing the translational relevance of the delayed myelination seen in mice to humans presents challenges. First, capturing precise information about myelin using MRI is difficult, and our current data do not directly measure myelination in humans. Second, histological validation in humans is not possible. Additionally, translating developmental data from rodent models to humans is challenging due to the inherent differences in timing across species. Future studies using multimodal imaging approaches could investigate whether and to what extent a delay in myelination also occurs in children with AS.

## Conclusions

In summary, our study shows that children with AS exhibit substantial WM and GM volume deficits, as we have previously shown for AS model mice, and demonstrate that these deficits are apparent as early as 1 year of life. Our studies in AS model mice suggest that brain volume reductions are accompanied by a delay in the onset of myelination. Further research is needed to determine whether these observed deficits and myelination delays are secondary consequences of AS or play a central role in its pathogenesis. Investigating these questions will improve our understanding of AS and aid in developing potential treatments. Additionally, our research shows that tracking changes in WM could be an effective method for evaluating the potential success of therapeutic interventions in AS patients, particularly for detecting early therapeutic responses in clinical trials.

## Figures and Tables

**Figure 1:** Developmental trajectory of brain volume in neurotypical and AS individuals. Representative axial, coronal, and sagittal orientations of MRI scans from (A) a 4-year-old neurotypical (NT) control and (B) a 4-year-old individual with Angelman syndrome (AS). Regions marked in yellow represent the segmented white matter (WM). (C) Quantification of total brain volume in NT controls and individuals with AS from 0.5 to 12 years of age. Quantification of (D) WM volume and (E) gray matter (GM) volume in NT controls and individuals with AS from 1 to 12 years of age. The boxplots show the (F) WM volumes and (G) GM volumes in NT controls and AS individuals from 6 to 12 years, corresponding to the age data points in dotted rectangles in (D) and (E).

**Figure 2:** Delayed myelination in AS mouse brain. (**A**) Representative Western blots for MBP and GAPDH loading control protein in P14 forebrain lysates from littermate pairs of *Ube3a*^*m−/p+*^ (AS) and wild-type (WT) mice. (**B**) Quantification of Western blotting for total MBP content (sum of 21.5, 18.5, 17 and 14 kDa isoforms). The total MBP content is significantly reduced in the forebrains of AS compared to WT mice (WT n=10, AS n=10, unpaired two-tailed t-test, P=0.026). Quantification of Western blotting for individual MBP isoforms (unpaired two-tailed t-test, 21.5 kDa: P= 0.0002, 18.5 kDa: P= 0.06, 17 kDa: P= 0.005, 14 kDa: P= 0.25). (**C**) Representative Western blots for MBP and GAPDH loading control protein in P14 forebrain lysates from littermate pairs of paternal *Ube3a*-null model (*Ube3a*^*m+/p−*^, PNL) and WT mice. (**D**) Quantification of Western blotting for total MBP content (WT n=7, PNL n=8, unpaired two-tailed t-test, P =0.93). Quantification of Western blotting for individual MBP isoforms (unpaired two-tailed t-test, 21.5 kDa: P=0.6, 18.5 kDa: P=0.97, 17 kDa: P=0.6, 14 kDa: P=0.42). (**E**) Representative Western blots for MBP and GAPDH loading control protein in P45 forebrain lysates from littermate pairs of AS and WT mice. (**F**) Quantification of Western blotting for total MBP content (WT n=7, AS n=7, unpaired two-tailed t-test, P =0.412). Quantification of Western blotting for individual MBP isoforms (unpaired two-tailed t-test, 21.5 kDa: P=0.18, 18.5 kDa: P=0.47, 17 kDa: P=0.09, 14 kDa: P=0.67). Data are mean ± SEM.

**Figure 3:** MBP developmental profile in the WT and AS mouse brain. Immunofluorescence staining for MBP (black) and DAPI (blue) in sagittal brain sections of WT and AS mice at P5 through P23. Boxes highlight regions with notably reduced MBP staining in AS mice compared to WT mice. Scale bars = 1 mm.

**Figure 4:** Delayed myelination in the AS hindbrain. Immunofluorescence staining for MBP in the superior olivary complex of WT mouse and AS littermate at P8. Scale bars = 100 μm. SOC, superior olivary complex.

**Figure 5:** Delayed myelination in the AS cerebellar cortex. Immunofluorescence staining for DAPI and MBP in the cerebellar cortex of WT mice and AS littermates at P8, P12, and P28. Arrow shows MBP staining along an oligodendrocyte in the WT cerebellar cortex at P8. Scale bars = 20 μm. EGL, external granule layer; ML, molecular layer; PL, Purkinje cell layer; IGL; inner granule layer.

**Figure 6:** Delayed myelination in the AS midbrain. Immunofluorescence staining for MBP in the midbrain of WT mice and AS littermates at P8, P16, and P28. The arrowhead indicates active myelination, while the arrow points to premyelinating oligodendrocytes. Insets provide a close-up view of the areas indicated by the arrowhead and the arrow. Scale bars = 200 μm; inset = 20 μm. SCm, superior colliculus motor related; SCs, superior colliculus sensory related.

**Figure 7:** Delayed myelination in the AS CA1 hippocampus. Immunofluorescence staining for DAPI and MBP in the hippocampal CA1 region of WT mice and AS littermates at P16, P45, and P60. Scale bars = 50 μm. SO, stratum oriens; SP, stratum pyramidale; SR, stratum radiatum.

**Figure 8:** Delayed myelination in the AS dentate gyrus. Immunofluorescence staining for DAPI and MBP in the dentate gyrus of WT mice and AS littermates at P28, P45, and P60. Scale bars = 50 μm. GCL, granule cell layer; PML, polymorph layer.

**Figure 9:** Delayed myelination in the AS globus pallidus. Immunofluorescence staining for MBP in the globus pallidus of WT mice and AS littermates at P5, P8, and P10. Scale bars = 100 μm.

**Figure 10:** Delayed myelination in the AS corpus callosum. Immunofluorescence staining for MBP in the body and genu of the corpus callosum in WT mice and AS littermates at P16 and P30. Scale bars = 50 μm. Bcc, body of corpus callosum; Gcc, genu of corpus callosum.

**Figure 11:** Ultrastructure of the developing WT and AS corpus callosum. Representative electron micrographs of the corpus callosum body in (**A1**) a WT mouse and (**B1**) a AS littermate at P16. Representative electron micrographs showing examples of myelinated axons observed in (**A2, A3, A4**) a WT mouse and (**B2, B3, B4**) an AS littermate at P16. Representative electron micrographs of the corpus callosum body in (**C1**) a WT mouse and (**D1**) a AS littermate at P30. Representative electron micrographs showing examples of myelinated axons observed in (**C2**, **C3, C4**) a WT mouse and (**D2, D3, D4**) a AS littermate at P30. (**E**) and (**F**) Representative electron micrographs showing layers of compacted myelin around the axons shown in C4 and D4. Scale bars: (**A1, B1, C1, D1**) = 500 nm; (**A2–4**), (**B2–4**), (**C2–4**), (**D2,4**) =125 nm, (**E**), (**F**) = 50 nm.

**Figure 12:** Percentage of myelinated axons, axon size, and *g*-ratio in the developing WT and AS corpus callosum. Quantification of percent myelinated axons in body of corpus callosum (Bcc) in WT and AS mice at P16 and P30 (n=4 mice for each genotypic group, unpaired two-tailed t-test, P=0.01 for P16, P= 0.72 for P30). Data are mean ± SEM. Distribution of diameters of unmyelinated and myelinated axons in WT and AS mice Bcc at P16 and P30. Distribution of *g*-ratios of myelinated axons in WT and AS mice Bcc at P16 and P30.

## Data Availability

The authors will make the raw data supporting this article’s conclusions available upon request.

## Abbreviations

AS: Angelman syndrome
WT: Wild type
NT: Neurotypical
UBE3A: Ubiquitin protein ligase E3A
WM: White matter
GM: Gray matter
MRI: Magnetic resonance imaging
MBP: Myelin basic protein
PNL: paternal-null
SOC: Superior olivary complex
SCm: Superior colliculus motor-related
SCs: Superior colliculus sensory-related
CA1: Cornu ammonis1
SO: Stratum oriens
SP: Stratum pyramidale
SR: Stratum radiatum
GCL: Granule cell layer of dentate gyrus
PML: Polymorph layer of dentate gyrus
Bcc: Body of corpus callosum
Gcc: Genu of corpus callosum
EGL: External granule layer of cerebellum
ML: Molecular layer of cerebellum
PL: Purkinje cell layer of cerebellum
IGL: Inner granule layer of cerebellum
